# Assessment of Dynamic Properties of Variable Area Flowmeters

**DOI:** 10.3390/s21092917

**Published:** 2021-04-21

**Authors:** Mateusz Turkowski, Artur Szczecki, Maciej Szudarek, Krzysztof Janiszowski

**Affiliations:** 1 Faculty of Mechatronics, Institute of Metrology and Biomedical Engineering, Warsaw University of Technology, 02-525 Warszawa, Poland; mateusz.turkowski@pw.edu.pl (M.T.); artur.szczecki.dokt@pw.edu.pl (A.S.); 2 Faculty of Mechatronics, Institute of Automatics and Robotics, Warsaw University of Technology, 02-525 Warszawa, Poland; krzysztof.janiszowski@pw.edu.pl

**Keywords:** variable area flowmeters, rotameters, flow sensors, micro sensors, industry 4.0, mathematical modeling

## Abstract

In previous works, a non-linear equation describing variable area (VA) flowmeters in transient was presented. The use of a full nonlinear equation, despite giving accurate results, can be difficult and time-consuming and it requires having specific software and knowledge at one’s disposal. The goal of this paper was to simplify the existing model so that it could be used in applications where ease of use and ease of implementation are more important than accuracy. The existing model was linearized and simple formulae describing natural frequency and damping coefficients were derived. With these parameters, it is possible to assess the dynamic properties of a variable area flowmeter. The step response form can be identified and natural frequency and settling time can be estimated. The linearized model and the experiment were in reasonable agreement. The step response type was captured correctly for each of the six VA meter types. The error in the undamped natural frequency was not larger than 15%, which means that the VA meter sensor’s dynamic properties can be predicted at the design stage with sufficient precision.

## 1. Introduction

The rapid development of Industry 4.0 and the Internet of Things (IoT) has caused an increased demand for various kinds of sensors. Newman [1] projects that there will be more than 41 billion IoT devices by 2027, up from about 8 billion in 2019. Most of such devices include one or more sensors that collect data and information from the surrounding environment. These sensors measure or detect such quantities as temperature, pressure, humidity, moisture, sound, light, motion, acceleration, magnetic fields, gases or objects in proximity. One of the essential quantities is flow rate [2]. Considering the number of devices that rely on flow rate, one can expect that simple, rugged and cheap sensors will be of great interest for these purposes. 

Therefore, the right solution for the measurement of flow rate could be a variable area (VA) flowmeter (commonly called a rotameter) equipped with a device that converts the position of a float into any standardized signal. In [3,4], a simple remote readout method is proposed that consists of a linear variable differential transformer. Other sources mention the application of Hall sensors [5,6], magneto-resistive sensor matrices parallel to the tube [7] or capacitance transmitters [8]. 

The VA flowmeter is a rugged and economical flow measurement device that is widely used for both low and medium flow rates in various industries [9,10,11]. IoT applications require a simple sensor construction and suitable operational properties, as well as decent metrological properties. VA flowmeters are characterized by moderate accuracy at best. However, similarly to process control and automation systems, in IoT applications, dynamic properties can be just as or even more important than static performance. Another advantage for IoT devices is the low and nearly constant pressure drop across VA meters.

In [12], such parameters as settling time, nature of step response (periodic or aperiodic) and overshoot of VA meters were studied and optimization procedures were presented. An analytical model describing VA meter dynamics was developed and validated experimentally. However, the application of such a model is time-consuming and, during the optimization process, nonlinear equations must be solved hundreds of times or more. Moreover, it requires appropriate software.

The main goal of this work is to simplify the existing formulae for applications where ease of use and ease of implementation are more important than accuracy. It was achieved by transforming the transient equation for VA meters into a linear form. With the linearized equation, the dynamic properties of the VA meter, such as undamped natural frequency and damping coefficient, can be obtained with a simple calculator. With such information, the dynamic performance of a variable area flowmeter can be easily assessed. It can determine if the response of the flowmeter will be oscillatory or aperiodic. In the case of oscillatory response, one can check whether the natural frequency would interfere with possible flow pulsations, which could cause resonance and uncontrolled bouncing of the float. Moreover, the linearized equations can be used for guidance on which parameters should be modified to obtain desirable behavior from the meter. Knowledge of natural frequency and damping can aid in tuning the flow control loop in process control applications.

The presented mathematical model can facilitate the development of new, miniaturized versions of VA meters, particularly useful for IoT applications. The model can also be used for diagnostics, where the registered flowmeter response to step change is compared against the linearized model.

## 2. Materials and Methods

### 2.1. Basic Theory of VA Meters

This subchapter recalls the basic theory of VA meter operation and introduces the terminology used throughout the article. For more information on complete derivations, see [12].

**Figure 1:**
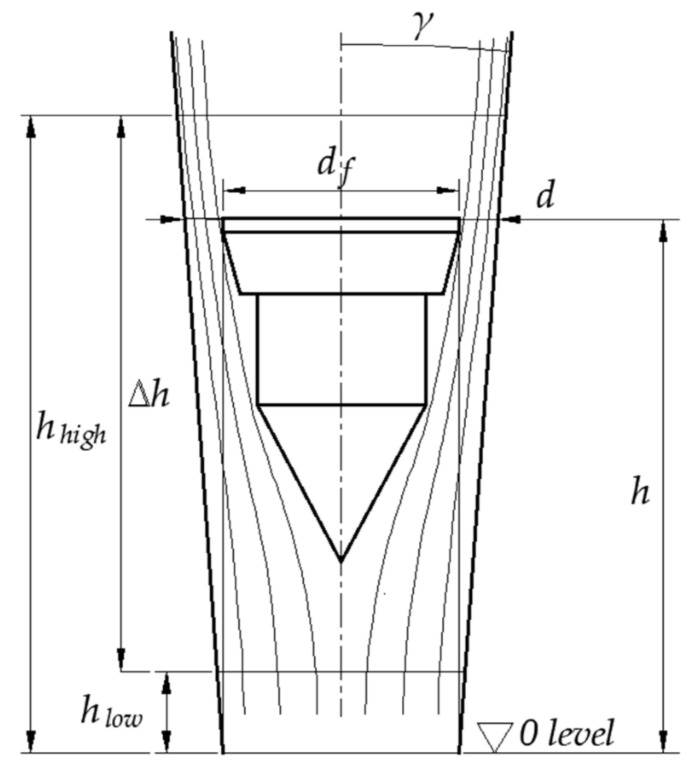
Simplified geometry of a variable area flowmeter and the streamlines across it (not to scale).

A simple VA meter’s crucial components are a conical tube and a float (Figure 1). Floats can be centrally guided, which may introduce friction effects or unguided, offering more repeatable results [13].

The pressure difference across the float results in an upthrust force balanced with the float’s immersed weight. Analysis of the force balance makes it possible to develop the steady-state equation of the VA flowmeter [12] in the form:

(1) $$Q=C\left(Re\right)A\sqrt{\frac{2gV_{f}\left(\rho_{f}-\rho \right)}{\rho A_{f}}}$$

where $A\left(h\right)=\pi \left(hd_{f}tan\gamma +h^{2}tan^{2}\gamma \right)$ is the area of the annular slot between the float and the tube, *d_f_* is the float diameter, *h* is the elevation of the float in the tube, *γ* is half of the tube’s tapering angle, *g* is the acceleration due to gravity, *V_f_* is the float volume, *ρ_f_* is the float density, *ρ* is the fluid density and $A_{f}=\pi d_{f}^{2}/4$ is the area of the perpendicular section of the float.

During the derivation of (1), numerous simplifying assumptions were made. The contraction effect is present, so the narrowest annular stream section, the “vena contracta”, is located at some point downstream of the float. Moreover, velocity profiles are not uniform and viscous effects together with dynamic drag take place. The secondary flows, usually appearing downstream bends or rectangular ducts [14], will also appear downstream of the float. The impact of all of these factors can be determined experimentally as a function of Reynolds number and introduced to Equation (1) in the form of the flow coefficient *C*(*Re*).

If volumetric flow rate changes over time, additional terms are present. The equation describing float movement for incompressible gas during transience was derived in [12] and it takes the following form:

(2) $$m\frac{d^{2}h}{dt^{2}}=\rho A_{f}\frac{{\left[Q\left(t\right)-C\left(Re\right)A\left(h\right)\frac{dh}{dt}-A_{f}\frac{dh}{dt}\right]}^{2}}{2{\left[C\left(Re\right)A\left(h\right)\right]}^{2}}-gm$$

where *m* is the mass of the float.

As the differential Equation (2) is nonlinear, it requires numerical methods to approximate its solution.

### 2.2. Transformation of the Equation of VA Meter into Linear Form

In [12], the optimization of the VA meter with the use of Equation (2) is presented. The use of a full nonlinear equation, although giving accurate results, was proven to be difficult, time-consuming and requiring specific software and knowledge at one’s disposal. During the optimization process, the equation must be solved hundreds of times or more.

From a practical point of view, it would, therefore, be advantageous to have simple formulae that allow quick computation of the natural frequency and damping of the VA flowmeter. In the case of a linear differential equation in the form:

(3) $$A_{2}\frac{d^{2}h}{dt^{2}}+A_{1}\frac{dh}{dt}+A_{0}h=BQ$$

it is possible to transform it using the Laplace transform into an algebraic equation:

(4) $$G\left(s\right)=\frac{Y\left(s\right)}{U\left(s\right)}=\frac{k{\omega_{0}}^{2}}{s^{2}+2\zeta \omega_{0}s+{\omega_{0}}^{2}}$$

where *k* is the static gain, $\omega_{0}$ is the natural frequency, *s* is a complex variable and *ζ* is the damping coefficient. Then, the natural frequency equals:

(5) $$\omega_{0}=\sqrt{\frac{A_{0}}{A_{2}}}$$

The damping coefficient *ζ* equals:

(6) $$\zeta =\frac{A_{1}}{2\sqrt{A_{0}A_{2}}}$$

and the damped natural frequency *ω* equals:

(7) $$\omega =\omega_{0}\sqrt{1-\zeta^{2}}$$

Formulae (5) and (6) may be the basis for optimization of the VA meter in terms of its dynamic performance (e.g., minimization of the settling time). The linearized version of Equation (2) is derived in Appendix A. The approach used assumes continuity of the function in Equation (3). Then, a Taylor series expansion in a stable operating point can be applied. This expansion is limited to first-order terms, as in [15]. The steady-state position of the float was chosen as the operating point.

The damping coefficient *ζ* and undamped natural frequency $\omega_{0}$ take the form:

(8) $$\omega_{0}=\sqrt{\frac{\rho A_{f}Q_{0}^{2}\left(\pi d_{f}tan\gamma +2\pi tan^{2}\gamma h_{0}\right)}{C\left(Re\right)A^{3}\left(h_{0}\right)m}}$$

(9) $$\zeta =\frac{\rho A_{f}\left[C\left(Re\right)A\left(h_{0}\right)+A_{f}\right]Q_{0}}{2\omega_{0}C\left(Re\right)A^{2}\left(h_{0}\right)m}$$

where *h*_0_ and *Q*_0_ are the position of the float in steady state and volumetric flow rate in steady state, respectively.

Then, the damped natural frequency *ω* can be calculated from Equation (7) and the height of the float in the VA meter can be calculated from:

(10) $$h\left(t\right)=kQ_{0}\left[1-\frac{1}{\sqrt{1-\zeta^{2}}}e^{-\omega_{0}\zeta t}sin\left(\omega_{0}\sqrt{1-\zeta^{2}}t+arctg\frac{\sqrt{1-\zeta^{2}}}{\zeta }\right)\right]$$

where $k=h_{0}/Q_{0}$ and *t* is time, counting from the flow rate step input.

### 2.3. Experimental Setup

Six VA flowmeters with two float shapes (plumb bob and sphere) were investigated. Meters were calibrated with the use of a bell prover and flow coefficient *C* was determined experimentally as a function of the Reynolds number. There was a question of the appropriate approximating function, which would allow the calculation of a flow coefficient for any Reynolds number. Various approximating functions were verified: first-, second- and third-order polynomials, exponential functions and logarithmic functions. The residual sum of squares was calculated to estimate the goodness of fit. The best fit was obtained with the use of a logarithmic function:

(11) $$C\left(Re\right)=Dln\left[E\cdot Re\left(t\right)+1\right]$$

Coefficients *D* and *E* are listed for each VA meter in Table 1. The averaged values of *D* and *E* coefficients for a plumb bob float can be used for the initial, rough approximation of the flow coefficient at the design stage, when the real experimental data are not yet known.

The experimental study concerning the dynamic performance of VA meters was conducted with a methodology fully developed by the authors, which is described in detail in [12].

**Figure 2:**
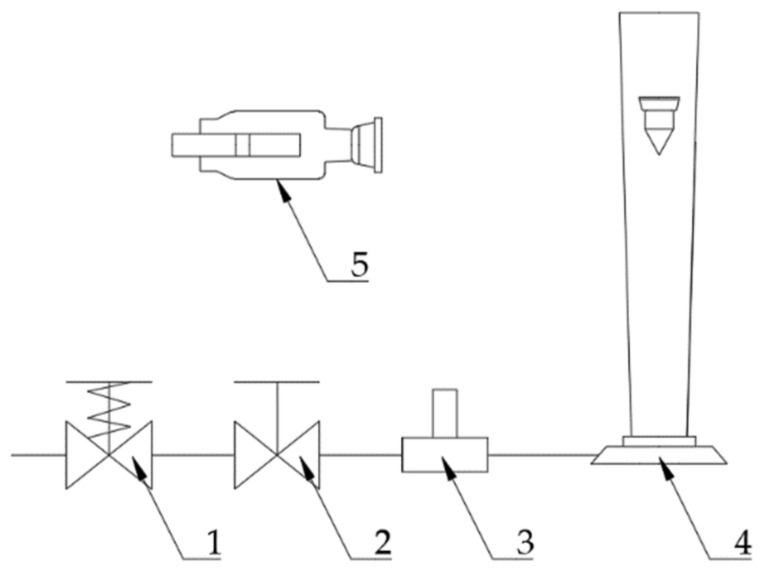
Test stand for the study of the VA meter’s dynamic performance. 1—fast-opening valve; 2—flow control valve; 3—constant-temperature anemometer with data acquisition system; 4—VA meter; 5—camera.

The test stand is shown in Figure 2. The step change in the flow rate was generated by opening a fast spring-operated valve. The volumetric flow rate step input was not ideal. Therefore, it was registered using a hot wire anemometer and later approximated by a function in the form of

(12) $$Q\left(t\right)=Q_{0}\left(1-e^{-90t}\right)$$

This formula was used in the nonlinear equation solution. In the case of the linearized equation, ideal step input was assumed.

The step response of the meter was registered with an analog 8 mm camera with a framerate of 50 frames per second. The position of the float was then measured on the screen of a film previewer.

### 2.4. Uncertainty Evaluation

Uncertainty analysis for steady state was developed in [12] under the assumption that the results will be comparable to the transient. As the experimental setup remains the same. the previously assessed uncertainty values apply to the present study as well.

The uncertainty of computing the steady-state position of the float varied from meter to meter. The largest uncertainty concerned the 3–30 dm^3^/h VA meter, where the expanded uncertainty was equal to *U*_95_(*h*) = 18.02 mm. The uncertainties were smaller for larger VA meters and were in the range of 2–4.5 mm.

The experimental uncertainty was assessed to be in the range of 0.4–2.6 mm.

## 3. Results and Discussion

There is a question of how such a massive simplification as linearization influences results. In this chapter, the linearized equation results are presented alongside the experimental data and the full nonlinear equation solutions.

**Figure 3:**
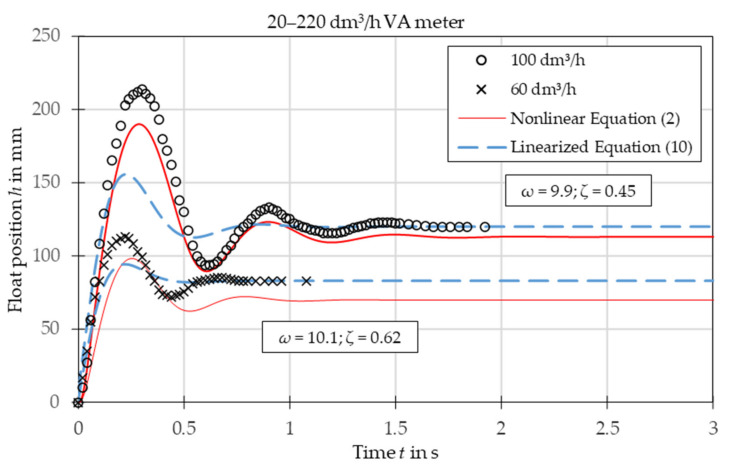
Step response for VA meter no. 2 for tested flow rates 60 dm^3^/h and 100 dm^3^/h. Damped natural frequency *ω* and damping ratio *ζ* refer to linearized equation solutions.

**Figure 4:**
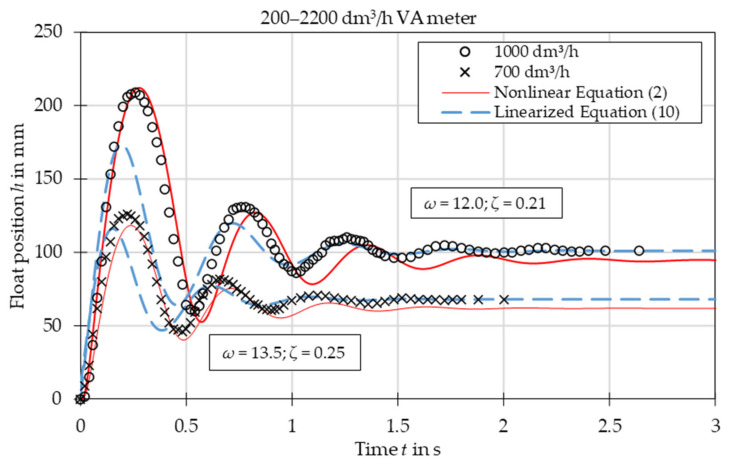
Step response for VA meter no. 4 for tested flow rates 700 dm^3^/h and 1000 dm^3^/h. Damped natural frequency *ω* and damping ratio *ζ* refer to linearized equation solutions.

**Figure 5:**
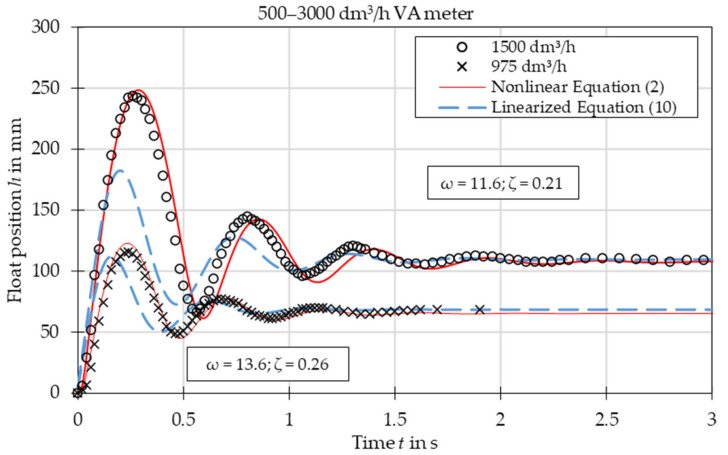
Step response for VA meter no. 5 for tested flow rates 975 dm^3^/h and 1500 dm^3^/h. Damped natural frequency *ω* and damping ratio *ζ* refer to linearized equation solutions.

**Figure 6:**
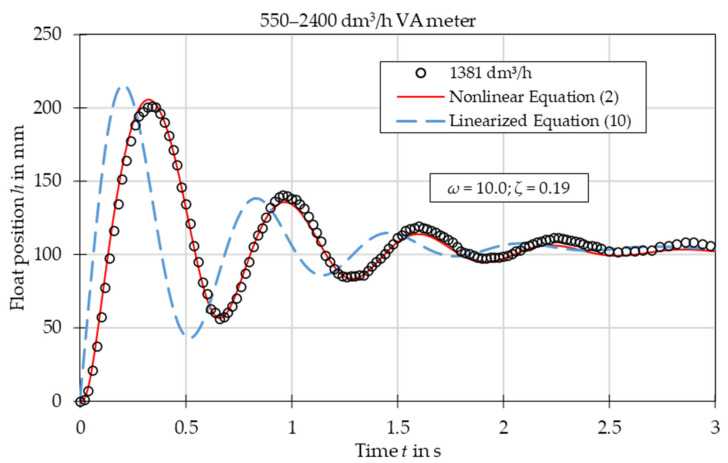
Step response for VA meter no. 6 for tested flow rate 1381 dm^3^/h. Damped natural frequency *ω* and damping ratio *ζ* refer to linearized equation solution.

**Figure 7:**
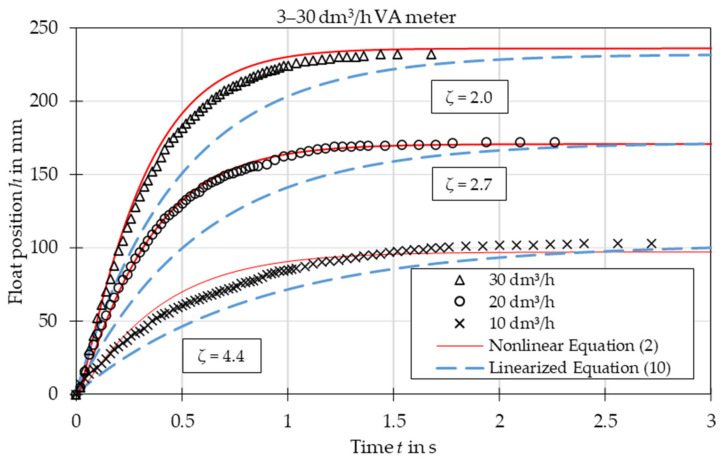
Step response for VA meter no. 1 for tested flow rates 10 dm^3^/h, 20 dm^3^/h and 30 dm^3^/h. Damping ratio *ζ* refers to linearized equation solutions.

**Figure 8:**
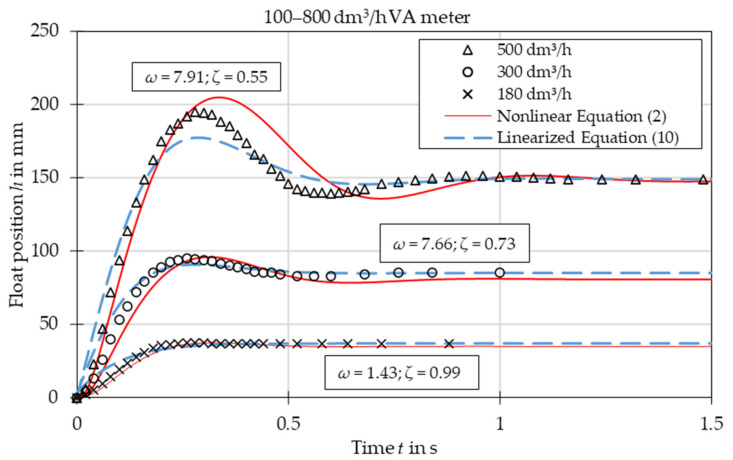
Step response for VA meter no. 3 for tested flow rates 180 dm^3^/h, 300 dm^3^/h and 500 dm^3^/h. Damped natural frequency *ω* and damping ratio *ζ* refer to linearized equation solutions.

**Figure 9:**
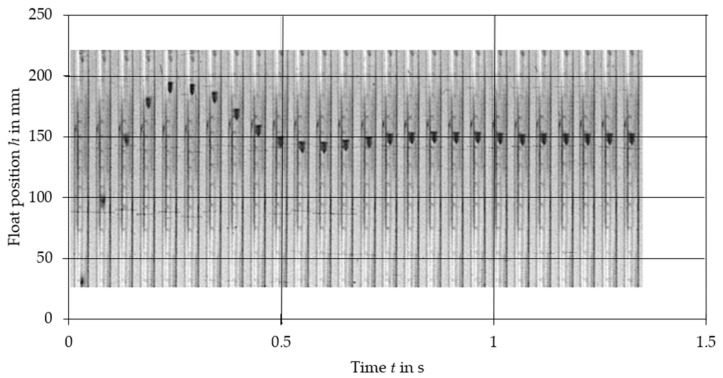
Selected frames from the 500 dm^3^/h step response of VA meter no. 3.

**Figure 10:**
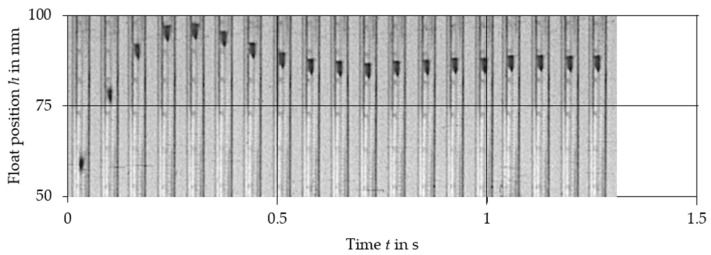
Selected frames from the 300 dm^3^/h step response of VA meter no. 3.

**Figure 11:**
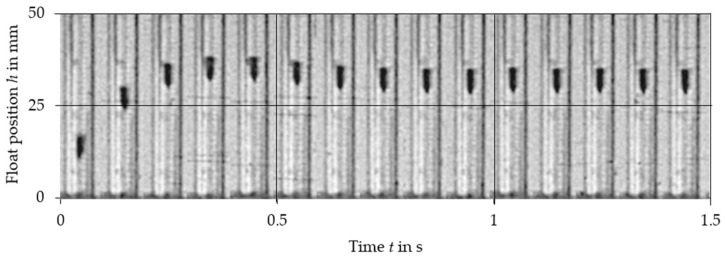
Selected frames from the 180 dm^3^/h step response of VA meter no. 3.

The comparison of experimental data with the results obtained from the nonlinear Equation (2) and linearized Equation (10) is presented in Figure 3, Figure 4, Figure 5, Figure 6, Figure 7 and Figure 8. Figure 9, Figure 10 and Figure 11 present selected images of a step response captured by camera for various step values. In [12], more images are present for other response forms. The experimental data match the solutions of the nonlinear equation very well. The differences between experimentally captured step responses and nonlinear equation solutions are discussed in detail in [12].

The analysis of the calculated values of natural damped frequency *ω* and damping coefficient *ζ* presented in Figure 3, Figure 4, Figure 5, Figure 6, Figure 7 and Figure 8 can be used to understand VA meter behavior.

The oscillation frequency was higher for a lower step value in Figure 3, Figure 4 and Figure 5. Moreover, oscillation fragments below steady-state value were shorter than the upper parts in Figure 3, Figure 4, Figure 5 and Figure 6. This is easy to explain if we notice that natural frequency also increased as step value decreased.

The settling time decreased with the step value in both aperiodic and oscillatory responses. This is also evident as the damping coefficient increased at lower step values. The overshoot in the oscillatory response was smaller for the linearized model and the settling time during the aperiodic response was longer.

Comparing parameters such as damping ratio is not possible, because in the experiment and the nonlinear model, the response of the system was significantly different from a linear response. To assess the differences between both models and experiments, parameters such as damped natural frequency, overshoot and rise time were calculated.

The mean absolute error of calculating the damped natural frequency with the linear model in comparison to the experiment was equal to 6%, with the largest errors not exceeding 15%.

Overshoot was compared for oscillatory responses. In the case of plumb bob floats, the linear model always underestimated the overshoot, on average by 32%. The full nonlinear model either under- or overestimated the overshoot with a mean absolute error of 23%. In the case of the spherical float, both models overestimated the overshoot—the linear model by 18% and the nonlinear model by 11%.

Rise time was compared for aperiodic responses. The mean absolute error was equal to 35% in the case of the linear model and 7% in the case of the nonlinear model.

The linearized model captured the step response type correctly for each of the studied cases. The accuracy of the linearized model was worse than that of the full nonlinear model. The largest discrepancies are seen in the Figure 3, especially in the initial stage of the float movement. This came from the fact that the linearization was based on one specific operating point—the steady-state position of the float. In the initial stage the float position was significantly different and the accuracy of the linearized model was reduced. The approximating function *C*(*Re*) was another reason for the discrepancies. Firstly, the flow coefficient itself is a simplification that considers float shape. The agreement between experiments and numerical simulations was better for the simple ball shape than for the more complicated plumb bob shape. Secondly, the approximating function *C*(*Re*) was obtained by means of best fit of experimental data. Moreover, there was the uncertainty of the experimental data and uncertainty related to the inputs to the model, such as the shape of the float and the tube, the form of the step function, etc. Another factor that influenced results was that the plumb bob float rotates which requires some energy from the flow. This fact was included by neither the nonlinear nor the linearized model.

Although the damping coefficient was overestimated by the linearized equation, it correctly represented the behavior of the VA meter during transience. If calculated for several flow values over the meter flow rate range, it could deliver valuable information regarding its dynamic properties.

## 4. Conclusions

The primary mathematical model [12] describing the VA meter float during transience in the form of a nonlinear equation was the basis of the work presented in this paper. The simplified model presented here could be applied in scenarios where ease of use and ease of implementation are more important than accuracy. It was successfully validated during laboratory experiments with air as the test medium, showing good agreement between the model and experimental results.

After linearization, simple and well-known parameters were determined, namely, the natural frequency *ω* and damping coefficient *ζ* of the meter. They allowed the identification of the form of the step response (oscillatory or aperiodic) and assessment of the settling time. Such a form is much more convenient for a system engineer or meter designer.

The accuracy of the linearized model was inevitably worse than the accuracy of the full, nonlinear model. For the plumb bob float shape, the mean absolute error in computed overshoot with the linear model was 32%, whereas, in the case of the nonlinear model, it was 23%. In the case of the spherical float, both models overestimated the overshoot, the linear model by 18% and the nonlinear model by 11%.

The rise time during aperiodic response was longer for the simplified model. The mean absolute error was equal to 35% in the case of the linear model and 7% in the case of the nonlinear model.

The natural frequencies calculated from the linearized model differed from the real values obtained from experiments by over 12%. However, both models reflected good agreement with the actual step response characteristics. The accuracy of data derived from the linearized model will, in most cases, be sufficient to predict the dynamic properties of the VA meter sensor at the design stage.

Further research directions could cover the dynamics of VA meters for liquids and other types of VA meters, such as orifice VA meters. The present paper is limited to plumb bob and sphere floats; the research scope could be extended to other float shapes in future.

## Figures and Tables

**Table 1:** List of investigated variable area (VA) flowmeters and their most important parameters.

VA No.	Nominal Range in dm^3^/h	Float Shape	*d_f_* in mm	*d_low_* in mm	*d_high_* in mm	Δ*h* in mm	*m* in g	*D*	*E*
1	3–30	Plumb bob	3.990	4.080	4.310	236	0.053	0.2369	0.0815
2	20–220	Plumb bob	4.030	4.130	4.560	223	0.381	0.2485	0.0388
3	100–800	Plumb bob	8.910	9.307	10.360	216	0.595	0.2132	0.0502
4	200–2200	Plumb bob	9.020	9.237	10.354	215	4.055	0.1789	0.0929
5	500–3000	Plumb bob	11.030	11.22	12.485	236	7.298	0.1444	0.297
Averaged for plumb bob								0.20438	0.11208
6	550–2400	Sphere	8.731	9.237	10.354	215	27.9	0.1680	0.1654

## Data Availability

Not applicable.

## Nomenclature

A = π (*d*^2^−*d**_f_*^2^)/4 (m^2^)	Area of the annulus between tube and float
*A*_0_. *A*_1_. *A*_2_. *B*	Coefficients of the differential equation
*A**_f_* = π*d**_f_*^2^/4 (m^2^)	Area of the float
*C* (-)	Flow coefficient
*d* (m)	Tube diameter corresponding to the current float position
*d**_f_* (m)	Float diameter
*d_low_* (m)	Tube diameter at the height corresponding to the lower flow rate limit *Q_min_*
*d_high_* (m)	Tube diameter at the height corresponding to the upper flow rate limit *Q_high_*
*D*, *E* (-)	Coefficients of a curve approximating *C*(*Re*)
*g* (m/s^2^)	Acceleration due to gravity
*h* (m)	Position of the float related to the zero level
*h*_0_ (m)	Position of the float in a steady state
*h_low_* (m)	Height corresponding to the lower flow rate limit *Q**_min_*
*h_high_* (m)	Height corresponding to the upper flow rate limit *Q**_max_*
*k* (m^2^/s)	Gain
*m* (kg)	Float mass
*Q* (m^3^/h)	Volumetric flow rate
*Q**_max_* (m^3^/h)	Upper flow limit
*Q**_min_* (m^3^/h)	Lower flow limit
*Q*_0_ (m^3^/h)	Volumetric flow rate in steady state
*Re* (-)	Reynolds number
*s* (-)	Complex variable
*t* (s)	Time
*U* (m/s)	Average fluid velocity
*U_95_* (-)	Expanded uncertainty at 95% confidence level
*V**_f_* (m^3^)	Float volume
Δ*h* (m)	Distance between *d**_low_* and *d**_high_*
*ν* (Pa·s)	Kinematic viscosity of the fluid
*ρ* (kg/m^3^)	Fluid density
*ρ**_f_* (kg/m^3^)	Float density
*γ* (rad)	Tapering angle
*ζ* (-)	Damping ratio
*ω* (1/s)	Damped natural frequency
*ω*_0_ (1/s)	Undamped natural frequency

## Appendix A

The known [12] transient differential equation of the VA meter (2) can be rewritten as

(A1) $$\begin{array}{c} 2{\left[C\left(Re\right)A\left(h\right)\right]}^{2}m\frac{d^{2}}{dt^{2}}h+\rho A_{f}\{2Q\left(t\right)\left[C\left(Re\right)A\left(h\right)+A_{f}\right]-[2C\left(Re\right)A\left(h\right)A_{f}+\\ {\left[C\left(Re\right)A\left(h\right)\right]}^{2}+{A_{f}}^{2}]\frac{d}{dt}h\}\frac{d}{dt}h+2{\left[C\left(Re\right)A\left(h\right)\right]}^{2}gm=\rho A_{f}Q{\left(t\right)}^{2} \end{array}$$

with

(A2) $$A\left(h\right)=\pi \left(hd_{f}tan\gamma +h^{2}tan^{2}\gamma \right)$$

Let us denote for convenience that $c=C\left(Re\right)$ $\beta_{1}=\pi d_{f}tan\gamma$ and $\beta_{2}=\pi tan^{2}\gamma$. Then,

(A3) $$A\left(h\right)=\beta_{1}h+\beta_{2}h^{2}$$

An introduction of space representation for this problem yields the following form:

(A4) $$x_{1}\left(t\right)=h\left(t\right)\;x_{2}\left(t\right)=\frac{d}{dt}h\left(t\right)$$

which allows representing the relation (A1) in the form

(A5) $$\begin{array}{ll} 2{\left[cA\left(x_{1}\right)\right]}^{2}m\frac{d}{dt}x_{2} & \\ & +\rho A_{f}\{2Q\left(t\right)[cA\left(x_{1}\right)+A_{f}]-[2cA\left(x_{1}\right)A_{f}+{\left[cA\left(x_{1}\right)\right]}^{2}\\ & +{A_{f}}^{2}]x_{2}\}x_{2}+2{\left[cA\left(x_{1}\right)\right]}^{2}gm=\rho A_{f}Q{\left(t\right)}^{2} \end{array}$$

After basic transformations. we obtain a state space representation:

(A6) $$\frac{d}{dt}x_{1}=x_{2}\;\begin{array}{ll} \frac{d}{dt}x_{2}=f\left(x_{1},x_{2},Q\right) & \\ & =-\frac{\rho A_{f}\left[cA\left(x_{1}\right)+A_{f}\right]}{{\left[cA\left(x_{1}\right)\right]}^{2}m}Q\left(t\right)x_{2}\\ & +\frac{2\rho A_{f}\{cA\left(x_{1}\right)A_{f}+{\left[cA\left(x_{1}\right)\right]}^{2}+{A_{f}}^{2}\}}{2{\left[cA\left(x_{1}\right)\right]}^{2}m}{x_{2}}^{2}-g+\frac{\rho A_{f}Q{\left(t\right)}^{2}}{2{\left[cA\left(x_{1}\right)\right]}^{2}m} \end{array}$$

where *X* = [*x*_1_(*t*) *x*_2_(*t*)]^T^ is a vector of states and *Q*(*t*) is an input vector. A linearization of nonlinear dynamic system (A6) in a considered state *X*_0_ yields a linear dynamic representation in the form:

(A7) $$\frac{d}{dt}X=AX+Bu\;\;\;u=Q\left(t\right)\;Y=CX\;\;\;y\left(t\right)=x_{1}\left(t\right)=h\left(t\right)$$

where

(A8) $$A=\left[\begin{array}{cc} 0 & 1\\ \alpha_{1} & \alpha_{2} \end{array}\right]\;\;\;B=\left[\begin{array}{c} 0\\ \lambda \end{array}\right]\;\;\;C=\left[\begin{array}{cc} 1 & 0 \end{array}\right]\;{\left.\alpha_{1}=\frac{d}{dx_{1}}f\left(x_{1},x_{2},Q\right)\right|}_{X_{0}}\;\;\;{\left.\alpha_{2}=\frac{d}{dx_{2}}f\left(x_{1},x_{2},Q\right)\right|}_{X_{0}}\;\;\;\lambda {\left.=\frac{d}{dQ}f\left(x_{1},x_{2},Q\right)\right|}_{X_{0}}$$

A transformation of (A7) into a transfer function form yields a compact representation of dynamics,

(A9) $$Y\left(s\right)=C{\left[s1-A\right]}^{-1}BU\left(s\right)\Rightarrow x_{1}\left(s\right)=\frac{\lambda }{s^{2}-\alpha_{2}s-\alpha_{1}}u\left(s\right)$$

that contains all of the basic information about the dynamic response of Δ*h*(*t*) in respect to flow Δ*Q*(*t*) changes:

(A10) $$H\left(s\right)=\frac{\beta }{s^{2}+2\zeta \omega_{0}s+{\omega_{0}}^{2}}Q\left(s\right)$$

where *β* is the gain in steady state. *ω*_0_ is the eigen-frequency value. and *ζ* is the damping factor. This representation must be developed for the steady state, when *h*(*t*) is constant and equal to *h*_0_. hence, *x*_2.0_ = 0.

The derivation of coefficients *α*_1_, *α*_2_ and *λ* for (A9) yields the following formula:

(A11) $$\begin{array}{ll} \alpha_{1}=\frac{d}{dx_{1}}f(x_{1},x_{2} & ,Q){|}_{X_{0}}\\ & =-{\left.\frac{d}{dx_{1}}\left\{\frac{\rho A_{f}\left[cA\left(x_{1}\right)+A_{f}\right]}{c^{2}A{\left(x_{1}\right)}^{2}m}Q\left(t\right)x_{2}\right\}\right|}_{X_{0}}\\ & +\frac{d}{dx_{1}}\{\frac{2\rho A_{f}c[A\left(x_{1}\right)A_{f}+{\left[cA\left(x_{1}\right)\right]}^{2}+{A_{f}}^{2}]}{2c^{2}A{\left(x_{1}\right)}^{2}m}{x_{2}}^{2}\}{|}_{X_{0}}\\ & +{\left.\frac{d}{dx_{1}}\left\{-g+\frac{\rho A_{f}Q{\left(t\right)}^{2}}{2c^{2}A{\left(x_{1}\right)}^{2}m}\right\}\right|}_{X_{0}} \end{array}$$

In the considered steady state *x*_2.0_ = 0; hence, it is not necessary to consider the first two terms of (A11) and in effect we obtain:

(A12) $$\begin{array}{ll} \alpha_{1}=\frac{d}{dx_{1}}f(x_{1},x_{2},Q){|}_{X_{0}}= & {\left.\frac{d}{dx_{1}}\left\{-g+\frac{\rho A_{f}Q{\left(t\right)}^{2}}{2c^{2}A{\left(x_{1}\right)}^{2}m}\right\}\right|}_{X_{0}}\\ & =-{\left.\frac{\rho A_{f}Q{\left(t\right)}^{2}\left[\beta_{1}+2\beta_{2}x_{1}\right]}{c^{2}A{\left(x_{1}\right)}^{3}m}\right|}_{X_{0}}=-\frac{\rho A_{f}{Q_{0}}^{2}\left[\beta_{1}+2\beta_{2}h_{0}\right]}{c^{2}A{\left(h_{0}\right)}^{3}m} \end{array}$$

In the case of the second coefficient *α*_2_, we can derive a similar formula, considering that terms with *x*_2_(*t*) will vanish at steady state:

(A13) $$\begin{array}{ll} \alpha_{2}=\frac{d}{dx_{2}}f(x_{1},x_{2}, & Q){|}_{X_{0}}\\ & =-\frac{d}{dx_{2}}\left\{\frac{\rho A_{f}\left[cA\left(x_{1}\right)+A_{f}\right]}{c^{2}A{\left(x_{1}\right)}^{2}m}Q\left(t\right)x_{2}\right\}{|}_{X_{0}}\\ & +\frac{d}{dx_{2}}\left\{\frac{2\rho A_{f}[cA\left(x_{1}\right)A_{f}+[cA\left(x_{1}\right){]}^{2}+A_{f}{}^{2}]}{2c^{2}A{\left(x_{1}\right)}^{2}m}x_{2}{}^{2}\right\}{|}_{X_{0}}\\ & +\frac{d}{dx_{2}}\left\{-g+\frac{\rho A_{f}Q{\left(t\right)}^{2}}{2c^{2}A{\left(x_{1}\right)}^{2}m}\right\}{|}_{X_{0}}=-\frac{\rho A_{f}\left[cA\left(x_{1}\right)+A_{f}\right]}{c^{2}A{\left(x_{1}\right)}^{2}m}Q\left(t\right){|}_{X_{0}}\\ & =-\frac{\rho A_{f}\left[cA\left(h_{0}\right)+A_{f}\right]}{c^{2}A{\left(h_{0}\right)}^{2}m}Q_{0} \end{array}$$

The coefficient *λ* representing a static gain (A9) is determined by a relation derived like others:

(A14) $$\begin{array}{ll} \lambda ={\left.\frac{d}{dQ}f\left(x_{1},x_{2},Q\right)\right|}_{X_{0}} & \\ & =-{\left.\frac{d}{dQ}\left\{\frac{\rho A_{f}\left[cA\left(x_{1}\right)+A_{f}\right]}{c^{2}A{\left(x_{1}\right)}^{2}m}Q\left(t\right)x_{2}\right\}\right|}_{X_{0}}\\ & +{\left.\frac{d}{dQ}\left\{\frac{2\rho A_{f}cA\left(x_{1}\right)A_{f}+{\left[cA\left(x_{1}\right)\right]}^{2}+{A_{f}}^{2}}{2c^{2}A{\left(x_{1}\right)}^{2}m}{x_{2}}^{2}\right\}\right|}_{X_{0}}\\ & +{\left.\frac{d}{dQ}\left\{-g+\frac{\rho A_{f}Q{\left(t\right)}^{2}}{2c^{2}A{\left(x_{1}\right)}^{2}m}\right\}\right|}_{X_{0}}={\left.\frac{2\rho A_{f}Q\left(t\right)}{2c^{2}A{\left(x_{1}\right)}^{2}m}\right|}_{X_{0}}\\ & =\frac{\rho A_{f}Q_{0}}{c^{2}A{\left(h_{0}\right)}^{2}m} \end{array}$$

Introducing values for steady-state point *h*_0_ and *Q*_0_, one can obtain the following results:

(A15) $$\omega_{0}=\sqrt{-\alpha_{1}}=\sqrt{\frac{\rho A_{f}{Q_{0}}^{2}\left[\beta_{1}+2\beta_{2}h_{0}\right]}{c^{2}A{\left(h_{0}\right)}^{3}m}}=\sqrt{\frac{\rho A_{f}{Q_{0}}^{2}\left[\pi d_{f}tan\gamma +2\pi {tan}^{2}\gamma h_{0}\right]}{c^{2}A{\left(h_{0}\right)}^{3}m}}$$

(A16) $$\zeta =\frac{-\alpha_{2}}{2\omega_{0}}=\frac{\rho A_{f}\left[cA\left(h_{0}\right)+A_{f}\right]}{2\omega_{0}c^{2}A{\left(h_{0}\right)}^{2}m}Q_{0}$$

(A17) $$k=\frac{\lambda }{\omega_{0}^{2}}≅\frac{h_{0}}{Q_{o}}$$
